# Calibration of Aseptic Loosening Simulation for Coatings Osteoinductive Effect

**DOI:** 10.1007/s10439-024-03588-9

**Published:** 2024-08-09

**Authors:** Sofia Baroni, Sara Oliviero, Antonino Amedeo La Mattina, Melania Maglio, Lucia Martini, Milena Fini, Marco Viceconti

**Affiliations:** 1 Department of Industrial Engineering, Alma Mater Studiorum - University of Bologna, Bologna, Italy; 2 Medical Technology Lab, IRCCS Istituto Ortopedico Rizzoli, Via di Barbiano1/10, 40136 Bologna, Italy; 3 SC Scienze e Tecnologie Chirurgiche, IRCCS Istituto Ortopedico Rizzoli, Bologna, Italy; 4 Scientific Direction, IRCCS Istituto Ortopedico Rizzoli, Bologna, Italy

**Keywords:** Osseointegration, Long-term stability, Bone-to-implant interface, In silico model, Micromotion, Finite element model

## Abstract

The risk of aseptic loosening in cementless hip stems can be reduced by improving osseointegration with osteoinductive coatings favoring long-term implant stability. Osseointegration is usually evaluated in vivo studies, which, however, do not reproduce the mechanically driven adaptation process. This study aims to develop an *in silico* model to predict implant osseointegration and the effect of induced micromotion on long-term stability, including a calibration of the material osteoinductivity with conventional in vivo studies. A Finite Element model of the tibia implanted with pins was generated, exploiting bone-to-implant contact measures of cylindrical titanium alloys implanted in rabbits’ tibiae. The evolution of the contact status between bone and implant was modeled using a finite state machine, which updated the contact state at each iteration based on relative micromotion, shear and tensile stresses, and bone-to-implant distance. The model was calibrated with *in vivo* data by identifying the maximum bridgeable gap. Afterward, a push-out test was simulated to predict the axial load that caused the macroscopic mobilization of the pin. The bone-implant bridgeable gap ranged between 50 μm and 80 μm. Predicted push-out strength ranged from 19 N to 21 N (5.4 MPa–3.4 MPa) depending on final bone-to-implant contact. Push-out strength agrees with experimental measurements from a previous animal study (4 ± 1 MPa), carried out using the same implant material, coated, or uncoated. This method can partially replace *in vivo* studies and predict the long-term stability of cementless hip stems.

**Supplementary Information:**

The online version contains supplementary material available at 10.1007/s10439-024-03588-9.

## Introduction

Total hip arthroplasty (THA) is one of the world’s most common orthopedic surgical procedures to treat hip osteoarthritis and hip fractures. The number of these procedures is continuously increasing [1–4] because of the increased life expectancy and increased number of younger patients undergoing hip joint replacement [2]. Therefore, it is crucial to continuously improve hip prosthesis devices to extend their life and reduce risks of failure. In particular, uncemented prostheses are becoming increasingly common compared to the past, being more suitable for younger patients than cemented ones [5]. Despite recent progress, cementless THAs are still associated with a significant revision rate (approximately 4-6% risk of failure at six-ten-year follow-up [6–8]), resulting in an average between OECD countries of 9 revision surgeries per 100.000 population in 2019 [4]. One of the leading causes of failure is aseptic loosening [9–12], i.e., the macroscopic instability of the femur prosthetic component with respect to the host bone in the absence of infection caused by lack of osseointegration. This is primarily initiated by inadequate implant mechanical stability (primary stability) from *press-fit* fixation. In this condition, daily physiological loading induces relative micromotion and causes the bone tissue at the interface to differentiate into fibrous, non-mineralized tissue, too weak to support the implant; the presence of fibrous tissue increases micromotion and results in a vicious loop, eventually producing a macroscopic instability of the stem, as well as pain under load. Pain is frequently severe, leading to revision surgery to replace the loosened stem. Therefore, long-term (secondary) stability is necessary to prevent implant mobilization. Secondary stability is achieved when the bone tissue adapts biologically and grows around the implant creating direct contact with it, i.e., osseointegration [13–15]. Aseptic loosening can be caused by different processes; however, focusing on contemporary cementless implants, where extreme stress shielding or massive wear is rarely observed, it is almost exclusively caused by excessive inducible micromotion linked to insufficient primary stability and to external loading. Therefore, an effective strategy to prevent aseptic loosening is to improve the implant coating from merely biocompatible to osteoinductive. An osteoinductive coating facilitates new mineralized tissue growth toward the stem surface, filling some gaps produced by reaming between the stem and the bone tissue [13–15]. This bone in-growth increases the initial bone-to-implant contact (BIC) surface and contributes to secondary stability. Therefore, aseptic loosening is a biomechanical and biochemical failure model.

Different preclinical in vivo and in vitro experimental settings are commonly used to study materials’ osseointegration and osteoinductive properties. The complexity of the osseointegration process, in fact, still requires the use of preclinical in vivo studies for evaluating the chemical-biological bone–implant interactions in vivo.

Osseointegration studies require implanting materials, usually cylindrical or screw shaped, in proper sites in cortical and/or trabecular bone. Depending on the animal model used, a minimum experimental time is defined for the evaluation of the interface between the material and bone tissue, the effective degree of osseointegration of the device, for example, with quantitative measures of bone surface in contact with the implant (Bone-to-implant contact—BIC), and the possible presence of biological phenomena related to the presence of the implant (formation of fibrous tissue, presence of inflammatory cells, etc.) [16–19]. As for pins, which are usually implanted in monocortical defects of the long bones diaphysis, physiological loads only induce negligible relative micromotion. Therefore, such experiments do not reproduce the mechanically driven adaptation process. In the past, *in vivo* experiments were used to evaluate how the osteoinductive properties of a coating can affect the tissue response to induced micromotion. For example, Søballe et al. [20] tested a micromotion device to induce movements during gait with implants coated with porous titanium or hydroxyapatite surrounded by a 750 µm gap. Each gait produced a micromovement of approximately 150 µm; after four weeks, histology analyses were performed comparing the tissue around these implants with the one surrounding a stable implant (without induced micromotion); in the first case, fibrous tissue was generated, while bone ingrowth was registered around the stable implant. Duyck et al. [19] designed a bone chamber implanted in rabbits’ tibiae to induce 30, 60, and 90 microns micromotion. They evaluated its effect on the BIC surface after six weeks.

The invasiveness and pain related to procedures involving devices to induce implant micromotion and the ethical and legal indication of developing alternative methods to reduce, refine, and replace in vivo models [21] require finding new experimental approaches. In fact, models without inducible micromotion, where a pin on the materials to be tested is firmly fitted in the cortical wall of the animal’s long bones, can provide information about the osteoinductive potential of a new coating type under optimal conditions, but they do not tell us what would happen when some inducible micromotion is present.

To overcome this limitation, *in silico* methods could be used to replace animal experiments after appropriate validation [22]. Patient-specific computer models were developed to predict primary and secondary implant stability for hip prosthesis stems [23–27]. Viceconti et al. [28] developed an interface remodeling simulation implemented as a finite states machine, where contact elements between bone and implant could change their status under the assumption that the relative bone–implant micromotion induced by loading was the main factor for long-term osseointegration. That model did account for all biomechanical factors, such as bone anatomy and quality, geometry and pose of the implant with respect to the anatomy, surface finishing, and external loading; however, the main limitation of that approach was that it did not account for the possible presence of osteoinductive coatings. Nowadays, most designs are coated with bioactive material, which, in principle, could enable bone ingrowth to fill more significant gaps. However, how large such gaps can be dependent on many factors; thus, only animal experimentation can provide specific information on the bioactivity of each coating.

This study aims to develop a phenomenological interface model of the osseointegration process, calibrated using in vivo experimental results obtained without inducible implant micromotion. Such osteoinduction model could then be added to the secondary stability model, which accounts for the inducible micromotion under physiological loading conditions. This represents an extension of the Viceconti *et al.* model, where the specific osteoinductive potential of the coating can be integrated, if present, in order to account for the biochemical aspect of the bone-to-implant interaction in addition to the mechanical component.

## Materials and Methods

In this study, a solid biomechanics model of the rabbit tibia implanted with two metallic biocompatible pins was developed and solved using the finite element (FE) method. The model replicated an in vivo experiment to study the material osseointegration. Experimental in vivo data were used to calibrate the model and determine the entity of the bridgeable gap filled with mineralized tissue during the 12-week experimental time. Subsequently, the calibrated model was used to simulate the effect of induced implant micromotion, thus evaluating the ability of the model to replicate *in vivo* osseointegration studies. To reduce the use of in vivo models, data and samples collected from other previous in vivo studies have been employed.

### Experimental Data

Osseointegration data at 12 weeks were collected from a previous in vivo study—performed according to the Italian Law on animal experiments (Law by Decree 26/2014), after the approvals of the research protocol by the Animal Welfare Body of IRCCS Istituto Ortopedico Rizzoli and by the Italian Ministry of Health, in which cylindrical titanium alloy implants (Ti6Al4V) of 2 mm diameter and 6 mm length were implanted in the tibiae diaphysis of four skeletally mature New Zealand male rabbits (Harlan Laboratories SRL, S. Pietro al Natisone, Udine) to investigate the material osseointegration ability.

At the end of the scheduled experimental time, samples were processed for methyl-methacrylate embedding as previously described [29]. Two histological sections (50 ± 10 µm) along the implant longitudinal axis were obtained for each implant to measure BIC percentage. Sections were stained with Stevenel Blue- and Picrofucsin [30]. BIC percentages were obtained by manual measurements on the histological images acquired with a digital scanner (Aperio ScanScope CS, Aperio Technologies, USA).

For collection of microtomographic images and BIC data at time zero, *ex vivo* implantation of the same titanium alloy implants was performed on rabbits’ tibiae collected as waste material at the end of another *in vivo* study, not affecting joints and femoral/tibial segments, conducted on animals of same strain and age. From the same images acquired at time zero, bone-to-implant distances were also measured in the areas where contact condition was not achieved. Distance between bone and implant was manually measured from the histological sections (25 measurements per section, distance between measurements of approximately 70 µm). The minimum and maximum measured distances were 19.02 µm and 315.00 µm (average of 67.69 µm and SD of 100.20 µm).

### Finite Element Model

**Fig. 1:**
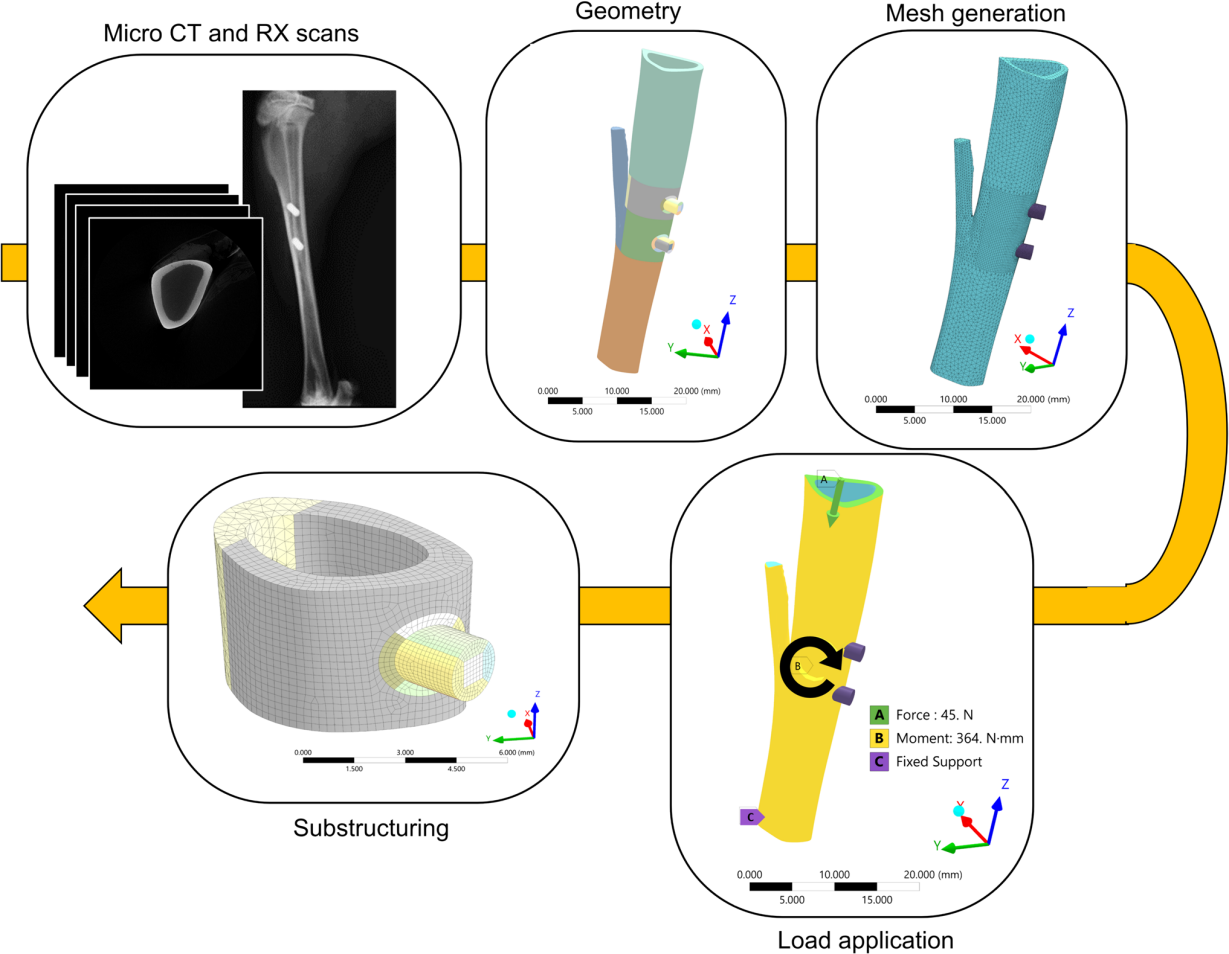
Model generation workflow from preclinical scans to FEM, using Mimics 24.0 (Materialise, Leuven, Belgium) and Ansys Workbench (Ansys release 2019 R3, ANSYS, Inc. Pennsylvania, USA)

**Fig. 2:**
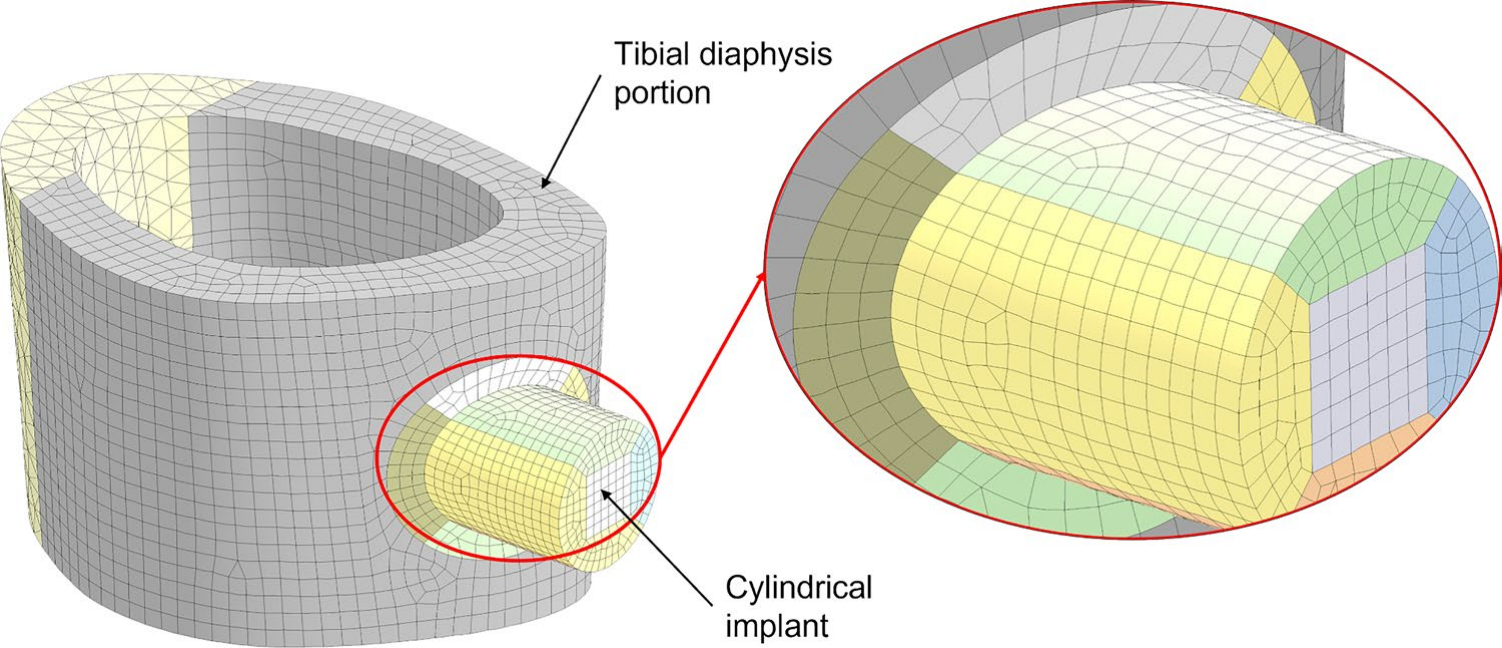
Contact region mapped mesh to each bidimensional implant element corresponds to one bidimensional bone element

A micro-Computed Tomography (micro-CT) image of the bone diaphysis from *ex vivo* implantation was obtained using a high-resolution micro-CT system (SkyScan 1172, Bruker, Belgium) with the following scanning parameters: source voltage 70 kV, current 141 µA, nominal resolution of 9.85 µm (pixel size), 885-ms exposure time, 0.50° rotation step, frame averaging of 3, and 0.5-mm Al filter (scan duration of about 31 min). The image was segmented using a global threshold (800 HU) in Mimics 24.0 (Materialise, Leuven, Belgium) and the bone volume imported in Ansys SpaceClaim (Ansys release 2019 R3, ANSYS, Inc. Pennsylvania, USA). Two cylindrical implants (2 mm in diameter and 6 mm in length) were inserted into the tibia with a Boolean subtraction, replicating the *in vivo* positioning observed in radiographic images (Fig. 1). The three components were divided into solids with shared topology to promote mesh generation. The FE mesh was generated using hexahedral quadratic elements (max element size = 0.25 mm) for the implants and the bone regions around implants, while tetrahedral quadratic elements (max element size = 0.62 mm) were generated at the proximal and distal ends of the tibia (Fig. 1). Contact regions were modeled with a mapped mesh made of bidimensional quadrilateral elements (Fig. 2). A Pure Penalty method was used for the contact problem, while the friction coefficient between bone and implant was initially set at 0.4 [31–33]. The model obtained contained 89082 elements and 222901 nodes. Material properties were assigned in Ansys Workbench: a titanium alloy from the software libraries (Young’s modulus = 96 GPa, Poisson’s ratio = 0.36) to the implants and average rabbit cortical bone properties (Young’s modulus = 10950 MPa, Poisson’s ratio = 0.3 [34, 35]) to the tibia. To the best of the authors’ knowledge, the available literature on the material properties of rabbit bone is limited. Since only the diaphyseal cortical bone was modeled, bone was simplified as a homogeneous material. This was considered acceptable as the variability observed with nanoindentation tests at the mid-diaphysis of rabbit long bones is relatively low [36]; additionally, homogeneous FE models have been validated against experimental measurements of local strains [34].

As boundary conditions, a compressive load (45 N) in the longitudinal direction and a bending moment (364 Nmm) in the mediolateral direction were applied to reproduce physiological loads as reported for the rabbit during running [34, 37, 38]. The distal end of the tibia was constrained with fixed support (Fig. 1). After the first simulation with the whole tibia volume, a smaller portion of the tibia containing the proximal implant was used for subsequent simulations (Fig. 1). Boundary conditions were assigned at the proximal and distal surfaces by applying the node displacements obtained by solving the whole tibia model. Therefore, the model was replaced by a smaller, more manageable, and less computationally expensive substructure containing the region of interest of this study: the BIC region (Fig. 1). The final model comprised 26837 elements and 79091 nodes, among which 376 bidimensional elements (max element size = 0.16 mm) and 1222 nodes constituted the contact region.

### Finite State Machine

The finite state machine model was based on the one described in Viceconti et al. [28], briefly summarized here. Three different types of contact elements were defined:

- *Bonded*: contact and target elements perfectly glued together, representing osseointegration.
- *Standard*: BIC regions that are not yet osseointegrated (Normal penalty stiffness factor FKN = 1, Friction coefficient = 0.4).
- *Fibrous*: elements with a very low contact stiffness (FKN = 0.001, Friction coefficient = 0.3), representing lack of osseointegration due to the formation of fibrous tissue following relative micromotion. Lower contact stiffness is used to simulate the presence of soft tissue by allowing penetration of the implant toward the bone surface.

In the model proposed by Viceconti [28], relative micromotion, shear, and tensile stresses between contact elements were used to determine and update each element's state at each subsequent iteration. Micromotion below a threshold of 40 µm determined *bonding*, while elements with micromotion above 150 µm became *fibrous*. Thresholds were chosen based on a review of previous osseointegration studies on different animal models, where induced displacement was applied to the implant with respect to the bone [39]. Osseointegration was observed when micromotion between bone and implant was lower than 40 µm [40, 41]. In contrast, the formation of fibrous tissue around the implant with very low contact stiffness was found when the imposed micromotion was about 150 µm [20, 39, 41–46]. *Bonded* contact elements returned to *standard* state if their shear stress exceeded 4.9 MPa or tensile stress exceeded 0.8 MPa, based on animal studies where push-out tests were performed on bone–implant interfaces previously osseointegrated [20, 46–49].

In this study, the model proposed by Viceconti et al. [28] was modified by adding a *gap* parameter representing the distance (gap) between implant and bone in the areas where BIC is not present to simulate that over time, small gaps can be filled by mineralized tissue, while larger gaps are not bridgeable. Therefore, a threshold was introduced for the bone–implant gap value above which *standard* and *bonded* states are never achieved, taking into account that bone has a limited ability to bridge the distance from the implant initially filled by soft tissue, also depending on the implant material or coating [47, 50–56]. *In vivo* data was used to calibrate the model to identify this threshold value and, subsequently, the ingrowth speed of the bone for the pin material used in this study, assuming that 12 weeks were sufficient to reach asymptotic behavior [57–59].

The measured distances between bone and implant (paragraph 2.1) were fitted using a Birnbaum–Saunders distribution with a median of 80.2 µm, a variance of 5191 µm^2^ and a mean value of 100.2 µm (fitdist function, Matlab R2022a, MathWorks, USA). Therefore, the initial conditions in the FE model were implemented by assigning the *standard* state (no gap) to randomly distributed elements according to the four initial BIC values (8.2%, 8.85%, 9.28%, 11.8%) measured experimentally. *Fibrous* state was assigned to the remaining elements, with different *gap* values obtained by sampling the fitted distribution (with 10-µm binning). Gap values were assigned to each element using a specific Contact Surface Offset (CNOF element *Real Constant*, Ansys). Hence, the initial contact configuration observed in the *in vivo* study was replicated in terms of both BIC area and bone–implant distances.

### Simulation of Osseointegration

**Fig. 3:**
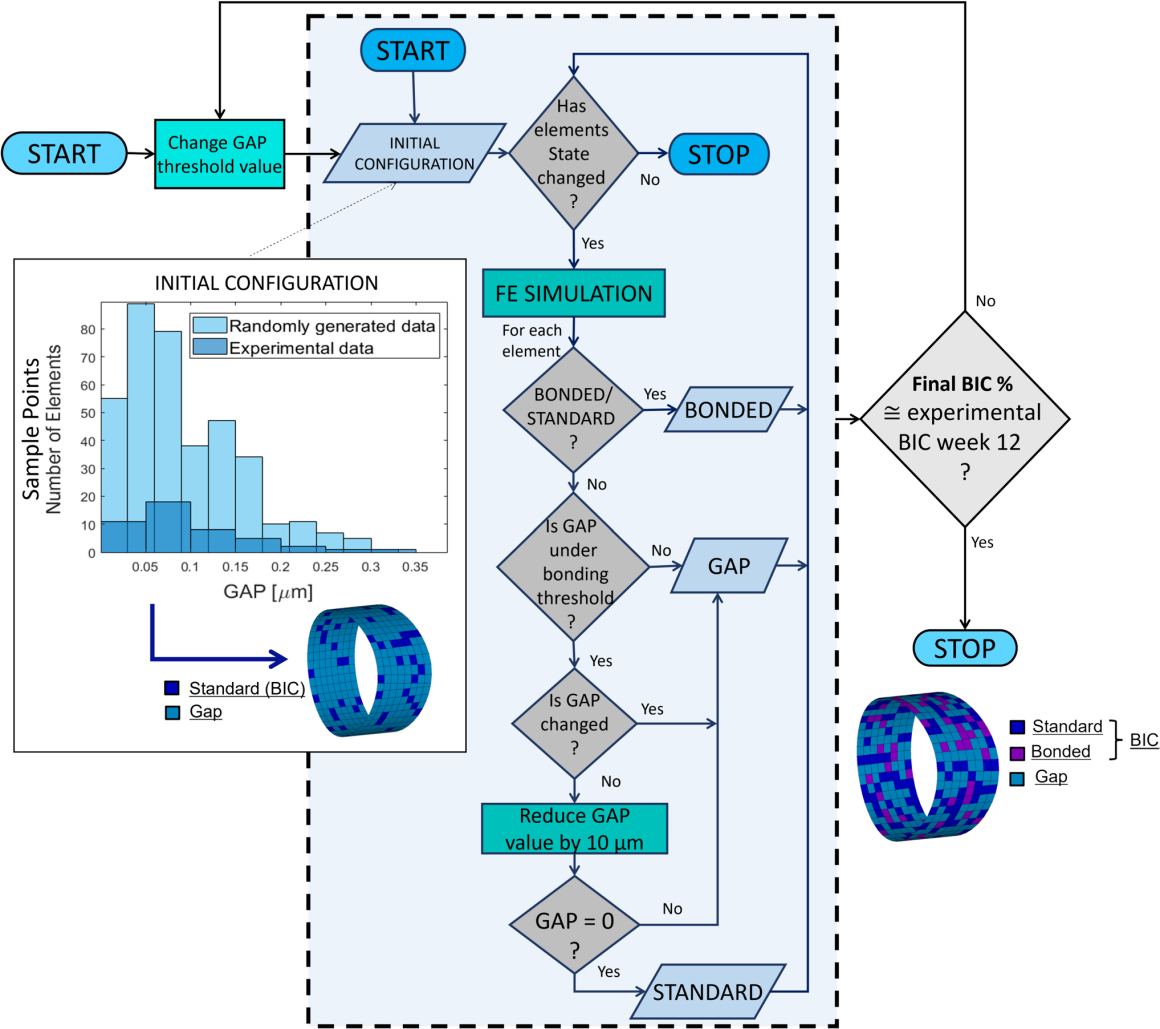
Osseointegration process implemented as finite state machine. Experimental BIC and gap distribution are used to define initial contact configuration. Gap data are randomly generated from the distribution obtained by fitting the experimental data (Birnbaum–Saunders distribution). Spatial configuration of this distribution is represented on the contact surface, where each color indicates a different gap value. The finite state machine runs each gap threshold value until convergence. A bridgeable gap is obtained when the final BIC matches the experimental value

The simulation of the osseointegration process was carried out, as represented in Fig. 3, and used to determine the maximum gap the bone could bridge to achieve BIC. Contact elements (*standard* state) became *bonded* at every iteration if the relative micromotion was lower than 40 µm. While *bonded* contact elements became *standard* if, at the end of the previous iteration, shear and tensile stresses were above the mentioned thresholds (paragraph 2.3), simulating the failure of bone bridges. For each gap element, the bone–implant gap value was updated after each iteration if it was lower or higher than in the previous one, representing the formation of bone bridges. The process was repeated using different gap threshold values (from 50 µm to 110 µm based on gap ranges from previous studies [52] and considering the implant roughness of 6 µm), iterating the algorithm until convergence. Convergence was defined as the stability of elements states. The maximum bridgeable gap was determined as the gap that reproduced the final BIC values (38.5%, 52.9%, 57.5%, 60.1%) measured experimentally. Multiple simulations were conducted by varying the gap threshold until the measured BIC final configuration from the animal experiment was obtained. This value represents the maximum distance from the implant bridgeable by the bone in 12 weeks, given the initial BIC configuration. The final BIC from the FE model was measured by including bonded and standard states elements. Preliminary simulations showed that the (small) variability in initial BIC experimental values does not affect the gap threshold obtained at convergence. Therefore, all subsequent simulations used the average BIC at time zero as the initial configuration.

### Simulation of Induced Micromotion

**Fig. 4:**
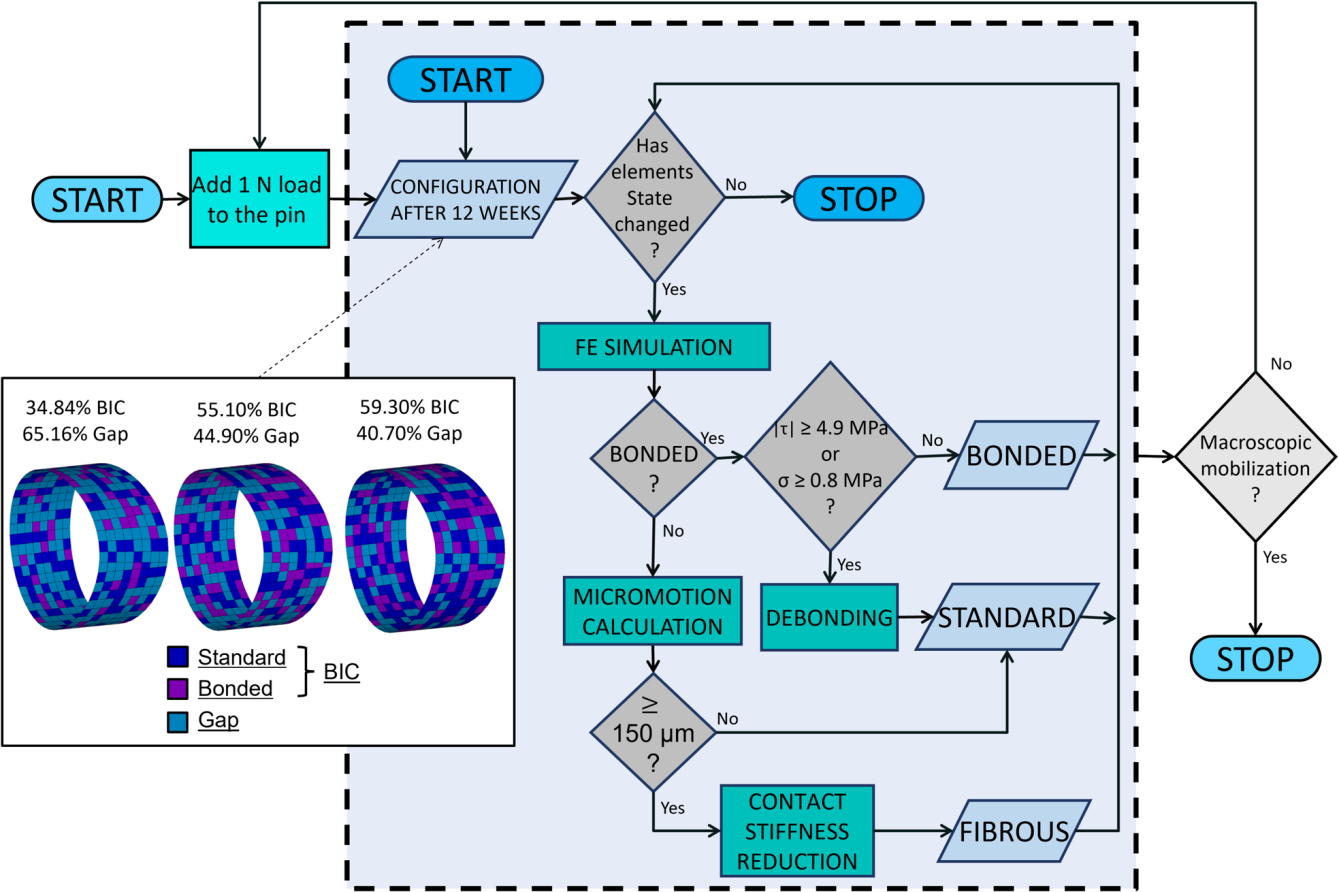
Induced micromotion implementation. Initial configuration is defined as BIC surface configuration obtained at the end of the previous simulation used for model calibration. The finite state machine is run until convergence for each induced micromotion value is applied. Macroscopic mobilization is defined as the transition of all contact elements to a fibrous state

The calibrated model obtained was used to investigate the effect of induced micromotion and identify the load causing macroscopic mobilization of the pin. The final configurations obtained from the previous study were used as input for this second simulator. An axial static load was applied to the pin in the mediolateral direction. For these simulations, the re-bonding process at each iteration was suspended to simulate that in the time frame of a push-out test and in the presence of a sufficiently high force applied to the implant, osseointegration is prevented [60–62]. Thus, elements in *standard* state did not become *bonded* even if relative micromotion was lower than 40 µm. When *bonded* elements de-bonded (shear and tensile stressed exceeding the thresholds) and assumed *standard* state, their friction coefficient was modified to 0.8 to simulate the rough surface generated by the bone bridge fracture. As previously described, *standard* elements with relative micromotion of 150 µm or higher became *fibrous* tissue that prevents the bone from stabilizing the implant. Using the finite state machine updated as described, different load values were applied to the pin from 1N to 21 N (increasing by 1N at each simulation) to identify the load that determined macroscopic mobilization, defined as the transition of all contact elements to *fibrous* state. When this occurs, the pin is surrounded by soft tissue, which causes its macroscopic mobilization. In the simulations, macroscopic mobilization is identified as lacking convergence due to rigid body motion. Bonded elements and/or friction are no longer sufficient to keep the pin stable. Therefore, the push-out load was identified as leading to non-convergence. The load for macroscopic mobilization was divided by the BIC area to calculate the push-out strength (Fig. 4).

### Sensitivity Analysis

A sensitivity analysis was performed using Cotter’s method [63] to evaluate the influence of model parameters and inputs on the model outputs. Briefly, this consisted in assigning to each parameter at a time its lower or upper value while keeping all others constant at their upper or lower values and evaluating the corresponding changes in the model output. This procedure allows to identify the key parameters that drive the model’s behavior. A first sensitivity analysis was run for the osseointegration simulation (paragraph 2.4) to evaluate the influence of input parameters on the final %BIC, percentage of *standard* and of *bonded* contact surface. Subsequently, a second analysis was carried out for the induced micromotion simulation (paragraph 2.5) to quantify their influence on the push-out strength. Input variables included in the analysis and their corresponding range of values are shown in Table 1.

**Table 1:** Input parameters, lower and upper value, and chosen value for the simulations

Parameters	Lower level value	Upper level value	Actual value	References	Output final BIC	Output push-out strength
Bonding micromotion threshold [µm]	30	50	40	[44]	X	
Fibroting micromotion threshold [µm]	140	160	150	[46]		X
Debonding tensile strength [MPa]	0.7	0.9	0.8	[48]	X	X
Debonding shear strength [MPa]	1.8	7.8	4.9	[49]	X	X
Gap interval [µm]	5	10	10	–	X	
Friction coefficient	0.2	0.6	0.4	[32, 33, 64, 65]	X	X
Initial BIC [%]	8.2	11.8	9.53	Experimental data	X	
Young’s modulus cortical bone [MPa]	10000	20000	10950	[34, 35]	X	

## Results

**Fig. 5:**
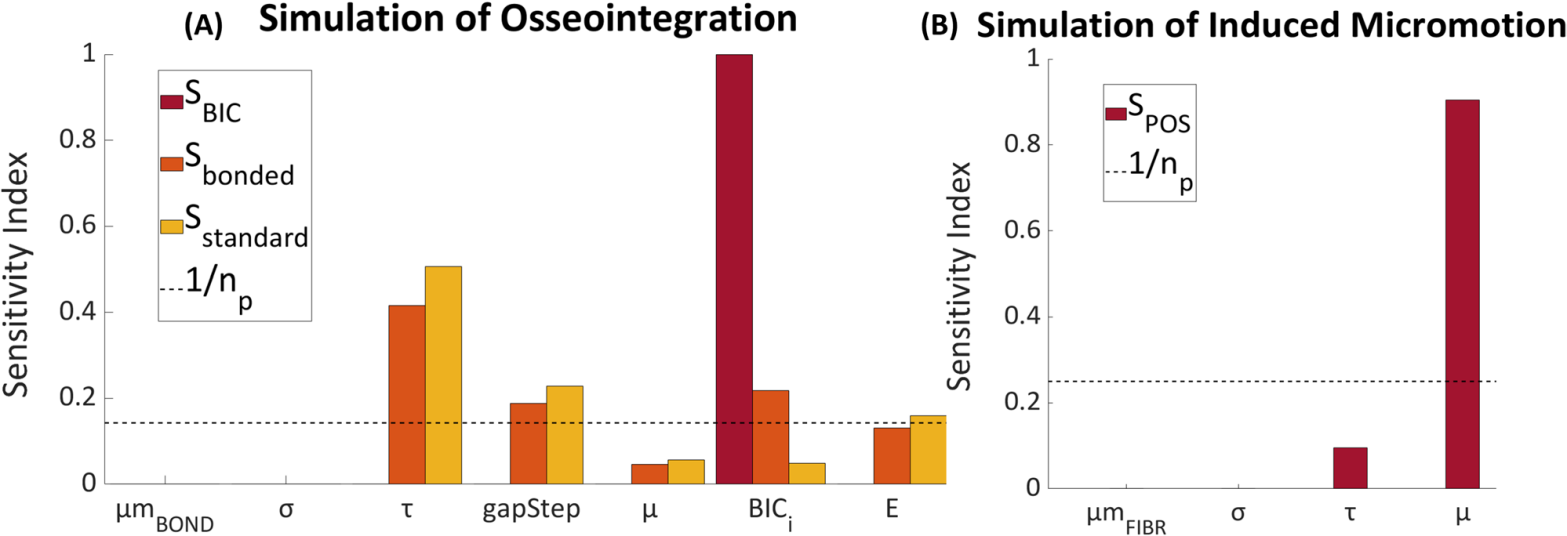
A Sensitivity indices for the osseointegration simulation. Output parameters: final BIC surface (*S*_BIC_), percentage of contact surface *bonded* (*S*_bonded_), and *standard* (*S*_standard_). Input parameters: bonding micromotion threshold (µm_BOND_), debonding tensile contact strength (*σ*), debonding shear contact strength (*τ*), gap discretization step (gapStep), friction coefficient (*µ*), initial BIC (BIC_i_), and bone elastic modulus (*E*). B: Sensitivity indices for the induced micromotion simulation. Output parameter: push-out strength (*S*_POS_). Input parameters: fibroting micromotion threshold (µm_FIBR_), *σ*, *τ*, and *µ*. Number of parameters = *n*_p_

Results of the sensitivity analyses are reported in Fig. 5. It was found that the initial BIC parameter had the most significant impact on the final BIC output. Final BIC varied in the range of 49.2% to 57.51% (Supplementary information 1). Debonding shear contact strength had the greatest impact on the final *bonded* and *standard* contact surfaces (*bonded* between 14.2% and 30.1% and *standard* between 19.2% and 43.4%). On the other hand, variations in the bonding micromotion threshold and debonding tensile contact strength had negligible effects on the outputs. Although friction coefficient variations had a low impact on the osseointegration simulation outputs, it significantly affected the push-out strength. Full details on output variations for each set of input parameters are reported in the Supplementary Information.

**Fig. 6:**
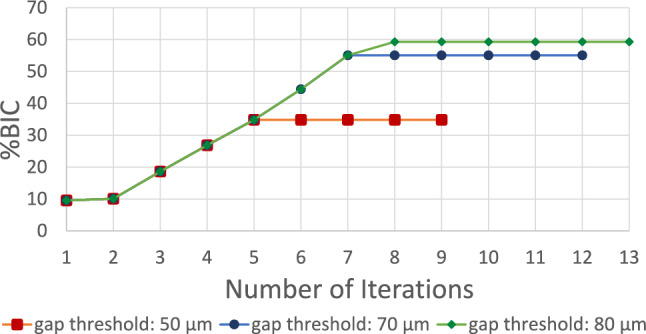
Convergence plots of the Finite State Machine calibration. BIC percentage after 9, 12, and 13 iterations with gap threshold, respectively, of 50 μm, 70 μm, and 80 μm

A monotonic convergence behavior was observed for the calibration of the finite state machine (Fig. 6). BIC increased over time until all elements with an initial gap under the defined threshold were in contact. The number of iterations to convergence varied (9–13 iterations) depending on the gap threshold value. Gap thresholds (maximum amount of bone–implant gap that bone was able to bridge in 12 weeks after implantation) were in the range of 50 to 80 μm depending on the final BIC (Table 2).

**Table 2:** Gap thresholds obtained from the model calibration for different final BIC values; push-out load and strength obtained with induced micromotion. Initial BIC is the mean of initial BICs obtained from the experimental study

Initial BIC (%)	Final BIC (%)	Gap threshold (µm)	BIC area (mm^2^)	Push-out load (N)	Push-out strength (MPa)
9.53	34.8	50	3.52	19	5.40
9.53	55.1	70	5.53	20	3.60
9.53	59.3	80	6.20	21	3.40

Push-out loads and strength obtained by inducing implant micromotion are reported in Table 2 for each final experimental configuration.

Push-out load was in the range of 19-21 N, corresponding to a push-out strength of 3.4-5.4 MPa.

## Discussion

The aim of this study was to develop an in silico model to simulate in vivo osseointegration studies. In particular, the model replicated the implantation of cylindrical implants in the rabbit tibia, a common osseointegration in vivo model. A previous [28] computational osseointegration model was extended to take into account, for further applications, the osteoinductive potential of the employed implant-specific coating by calibrating the interface model under physiological loading conditions using in vivo experimental results without inducible micromotion. The calibrated osteoinduction model was applied to a secondary stability prediction model, which simulates the application of micromotion to the implant.

The first model was used to calibrate the maximum bridgeable gap with experimental data obtained at 12 weeks after implantation of a titanium alloy pin, which showed that bone could fill a distance up to 80 μm from the implant. This can be considered consistent with previous experimental results where the reported bridgeable gap was smaller than 250 μm [52, 66], although, given the variability in bone types and experimental conditions, a direct comparison should be taken with caution. The bridgeable distance is expected to increase with osteoinductive coatings [67–69], and a similar procedure can be applied to consider this increased ability.

The maximum bridgeable gap and, therefore, the osseointegration capacity can vary depending on factors, such as bone quality, individual physiological conditions, environmental conditions, site of implant, implant dimensions and shape, and implant position with consequent physiological loading conditions [70]. However, a key factor is the presence of a porous or rough surface or a coating material to enhance implants’ biocompatibility and stimulate bone regeneration. The osteoinduction model presented in this study is calibrated using conventional experimental data. Therefore, while in this study an uncoated implant was modeled, this method allows to account for osteoinductive coatings, if present.

There is relatively little standardization of how osseointegration of metallic implants is investigated with animal models [71, 72]. One of the most critical aspects in developing in silico models starting from literature data is the limited histomorphometric analyses report data on the bone–implant gap and maximum bridgeable gap [71, 72], mostly published before 2000. Carlsson et al. investigated the critical gap between bone and pure titanium implants, which cannot be bridged by bone resulting in the formation of soft tissue, in a study on rabbit proximal tibia [52]. Pins of different diameters (2.0 mm to 3.7 mm) were implanted in fitting or larger bone defects (with gaps of 0, 350, and 850 μm). After 6 and 12 weeks, all the zero-gap implants had direct BIC. One 350-μm gap implant revealed direct bone apposition in the cortical area, while the others presented gaps (of 150 μm to 390 μm) after six weeks and gaps from 230 µm to 510 μm after 12 weeks. All 850-μm gap implants were surrounded by fibrous tissue. The authors concluded that a gap value of 350 μm is close to the critical gap width for direct lamellar bone apposition to implant surfaces. However, the lack of integration for most 350-μm gap implants suggests that this threshold is probably smaller. The main difference with the present study is that the bone-to-implant gap was uniform around the pin, while in this study, areas of contact and gap were not uniform around the implant. No data on smaller gaps were presented. Therefore, this study only confirms that a bridgeable gap is smaller than 350 μm. Other studies have evaluated final bone-to-implant distance as a function of the initial one, performed on different species and/or with different implants (i.e., threaded, coated, or made of different materials). For instance, Klein *et al.* investigated the minimum gap distance which a coated or uncoated implant cannot overbridge sufficiently by implanting in goats femurs titanium plugs (diameter: 5.1 mm, length: 7 mm) presenting four grooves each, respectively, 250 μm, 500 μm, 750 μm, and 1 mm deep [66]. After 3, 6, and 12 weeks of implantation, no groove was filled by bone ingrowth, with mostly zero BIC, concluding that the maximum bridgeable gap was smaller than 250 μm for uncoated titanium plugs. Mouzin *et al.* studied the effect of implant loading on bone growth in dog knee joints around a stable implant (6 mm diameter) with two different plasma-sprayed surfaces (titanium and hydroxyapatite) surrounded by a 750-μm gap [69]. After four weeks, the amount of bone volume measured for the titanium porous-coated implant in the gap zone far between 40 μm and 225 μm from the titanium implant surface was 11.4 ± 2.20%, probably meaning that the BIC was close to zero in this case. Another similar study [73] investigated the osseointegration of cylindrical titanium implants, plasma sprayed with a porous coating, with or without arginine–glycine–aspartic acid (RGD), implanted in eight skeletally mature mongrel dogs. For the non-RGD-coated implants, the critical gap was around 750 μm with the application of a loading [46] and 1 mm without loading after four weeks [74].

The push-out strength predicted in this study was comparable to that measured in a previous *in vivo* study conducted with similar cylindrical (2.8 mm in diameter and 6 mm in length) titanium implants coated or uncoated [60], inserted in the femoral cortical bone of fourteen rabbits. The measured push-out strength for pure titanium implants without coating was 4 ± 1 MPa after three months. Considering the bone type, follow-up time, pin material, and size, this study has the most substantial similarities with our experimental data. In another study, it was reported that for porous and rough cylindrical implants implanted in the cortical bone of rabbit tibiae, push-out strength was 3 MPa to 15 MPa after four weeks and 12 MPa up to 22 MPa after eight weeks, showing that porous and rough surfaces significantly improve implant fixation [75]. A lower push-out strength of 2 MPa was reported by Svehla et al. [76] for smooth titanium alloy pins (6 mm diameter, 15 mm length) implanted in the cortical bone of sheep tibiae. The lower push-out strength might be due, besides the different species, to the larger pin size used (greater contact area).

To our knowledge, this is the first application of a finite state machine finite element model to replicate and partially replace in vivo experiments, to inform a human model of osseointegration and improve orthopedic in silico models. The model developed by Viceconti et al. [28] was improved by introducing the bone–implant gap parameter to consider the time dependency of the osseointegration process using the data obtained from the in vivo model calibration. Previous studies have used finite element models of the hip stem osseointegration but used different methods to implement interface micromotions and gaps. For example, Tarala et al. [77, 78] implemented bone ingrowth simulation as a time-dependent process where non-bonded nodes are in frictional contact while bond between implant and bone nodes was modeled with springs of different stiffness. Bone ingrowth was a gradual increase of the local spring stiffness, the bonded condition was reached either when the local interface micromotion magnitude was lower than 40 μm and the local gap size did not exceed 1 mm depending also on local bone quality. Similarly, Chanda et al. [26] implemented the osseointegration process with an evolutionary change in the mechanical behavior of the interface modeled using spring elements connecting each interfacial implant node with the adjacent bone one. The spring elements stiffness value was updated iteratively from osseointegration level 1 up to 5 based on different rule-based criteria, such as interface gap lower than 150 μm, relative micromotion lower than 40 μm, compressive stress lower than 7.89 MPa, tensile stress lower than 0.80 MPa, and shear stress threshold based on literature data according to the osseointegration level. When a region reached the maximum osseointegration level, it was considered bonded condition.

A limitation of this study is the small number of available experimental measurements. Final BIC percentages were obtained from a single experimental study, which likely did not allow us to explore a wide range of BIC conditions or to fully assess the variability of the maximum bridgeable gap. Nevertheless, in this study, the computational model was successfully developed and calibrated with the experimental data, demonstrating the feasibility of the modeling approach and good agreement of the predicted results with, even though limited, literature data. Secondly, a number of parameters have been used to model the osteointegration process at the microscale, based on literature and previous studies, which could not be directly validated given the limited experimental data available. Sensitivity analyses were performed to assess their influence on the model outcomes (Results and Supplementary Information), which confirmed the robustness of the model predictions. In particular, the initial BIC had the greatest impact on the final BIC prediction, which can be expected as a larger initial contact area may favor osteointegration. When applying this approach to the simulation of a population of interest, a variability in initial BIC is expected; therefore, in future applications it could be modeled as a stochastic parameter, and be used to predict the inter-subject variability in final BIC. Similarly, a variability can be expected for debonding shear strength and friction coefficient, depending for example on the properties of the newly formed bone tissue. In future, these could be modeled as stochastic parameters as well, in order to statistically describe their variability in the population of interest. Nevertheless, it is worth mentioning that defining the appropriate distribution in the population is not trivial and would require appropriate experimental data. Additionally, it is important to highlight that the model remains phenomenological. For this application, the aim is not to accurately predict the osteointegration process at the microscopic level, but only its macroscopic manifestation, which drives the secondary stability and in turn the risk of aseptic loosening. The simulation iteration has no association with real time, while it represents the discretization step to reach asymptotic behavior, which phenomenologically simulates remodeling over time. Factors such as material surface biocompatibility and cell growth rate were not explicitly modeled, but their effect was taken into account only through the calibration with in vivo experimental data. Another limitation is that initial and final BIC cannot be measured longitudinally for the same rabbit. Therefore, subject-specific predictions were not obtained, and validation was limited to comparison with the experimental measurement range, although for different in vivo models, the contact configuration could vary significantly. Lastly, in this study, we only tested one pin material. A similar approach can be applied to predict osseointegration of different materials and/or with different coatings using similar experimental data from in vivo studies conducted without inducing micromotion on the implant. BIC at initial and final time points and gap measurements at the beginning of the study would be applied to the established pipeline for model calibration to evaluate bridgeable gaps for different coating materials and predict push-out strength.

The model proposed in this paper fits into the context of the expansion and integration of preclinical experimental modeling, in compliance with the principle of the 3Rs [79]. The aim is achieving an advance in alternative methods that allows the use of reliable simulations to reduce and replace where possible the in vivo model and design the in vivo studies in an optimized and more effective way.

The potential of this technology is that it can be employed for testing different prosthesis materials and coatings to predict long-term osseointegration, thus refining and partially replacing in vivo osseointegration experiments and predicting information not otherwise available from the in vivo tests. This model can be employed in future work to provide input data for human applications. The model may be integrated in FE models of the human femur for the prediction of long-term stability of cementless hip stems, where the osseointegration capacity of the material is taken into account as well as the bone–implant micromotion.

In conclusion, in this study, we have developed and calibrated an interface osseointegration model of in vivo studies to consider the material-specific osteoinductive potential. This modeling scheme predicted the implant push-out strength. It can be integrated into a human osseointegration model to predict the long-term stability of hip stems, representing an essential tool for new stem design.

## Supplementary Information

Below is the link to the electronic supplementary material.

Supplementary file1 (PDF 205 kb)

## Data Availability

Model data are available in the University of Bologna institutional repository under terms of CC BY 4.0 International license at the link 10.6092/unibo/amsacta/7813.
