# Supplementary material for: Calibration of Aseptic Loosening Simulation for Coatings Osteoinductive Effect
Source: Ann Biomed Eng. 2024 Aug 9;53(1):34–47. doi: 10.1007/s10439-024-03588-9 (PMC11782331; doi:10.1007/s10439-024-03588-9)
Supplement: Supplementary file 1 — Supplementary file1 (PDF 205 kb) [file 10439_2024_3588_MOESM1_ESM.pdf]

Supplementary Information

CALIBRATION OF ASEPTIC LOOSENING SIMULATION FOR COATINGS OSTEOINDUCTIVE EFFECT

Sofia Baroni[1,2], Sara Oliviero[1,2], Antonino Amedeo La Mattina[1,2], Melania Maglio[3], Lucia Martini[3], Milena Fini[4], Marco Viceconti[1,2]

[1] Medical Technology Lab, IRCCS Istituto Ortopedico Rizzoli, Bologna (IT)

[2] Department of Industrial Engineering, Alma Mater Studiorum - University of Bologna (IT)

[3] SC Scienze e Tecnologie Chirurgiche, IRCCS Istituto Ortopedico Rizzoli, Bologna (IT)

[4] Scientific Director, IRCCS Istituto Ortopedico Rizzoli, Bologna (IT)

CORRESPONDING AUTHOR:

Sofia Baroni

Medical Technology Lab

IRCCS Istituto Ortopedico Rizzoli

Via di Barbiano 1/10, 40136 Bologna (IT)

Email: sofia.baroni2@unibo.it

Supplementary information 1

A1. Sensitivity analyses

Sensitivity analyses were performed using Cotter's method [1] which allows to rank the input parameters' influence on the model outputs. This technique adopts a two-level fractional factorial design where all input parameter values are positioned at either the upper or lower bounds of their respective ranges. An initial simulation (y0, Table A1.1, and Table A1.2) is run where all parameters are set to their lower value, while for the last simulation all parameters are set to their upper level (y15 in Table A1.1 and y9 in Table A1.2). At each intermediate simulation, one parameter at a time is set to the minimum or maximum value while keeping other parameters at the opposite extreme. Two sensitivity analyses were performed, for the osseointegration simulation model and for the induced micromotion simulation model; the process required 16 simulations for the first and 10 simulations for the second. Output values obtained for each simulation of the sensitivity analyses are reported in Table A1.1 (osseointegration simulation) and Table A1.2 (induced micromotion simulation).

		Outputs		
**Observation**	**Factors at high level**	**Final BIC [%]**	***Bonded* surface [%]**	***Standard* surface [%]**
y0	-	49.2	13.56	35.64
y1	1	49.2	13.56	35.64
y2	2	49.2	13.56	35.64
y3	3	49.2	30.05	19.15
y4	4	49.2	16.22	32.98
y5	5	49.2	11.4	37.8
y6	6	57.51	20.01	37.5
y7	7	49.2	14.4	34.8
y8	2,3,4,5,6,7	57.51	33.57	23.94
y9	1,3,4,5,6,7	57.51	33.57	23.94
y10	1,2,4,5,6,7	57.51	14.16	43.35
y11	1,2,3,5,6,7	57.51	42.35	15.16
y12	1,2,3,4,6,7	57.51	34.64	22.87
y13	1,2,3,4,5,7	49.2	23.4	25.8
y14	1,2,3,4,5,6	57.51	39.69	17.82
y15	1,2,3,4,5,6,7	57.51	33.57	23.94

Table A1.1 Sensitivity analysis for the osseointegration model. Outputs obtained for each simulation (y0-y15) are reported. Factors 1 to 7 are set at either their lower or higher value. Factor 1: Bonding micromotion threshold; Factor 2: debonding tensile strength; Factor 3: debonding shear strength; Factor 4: gap interval; Factor 5: friction coefficient; Factor 6: initial BIC; Factor 7: Young's modulus of cortical bone.

		Outputs	
**Observation**	**Factors at high level**	**Push out force [N]**	**Push out strength [MPa]**
y0	-	12.5	2.26
y1	1	12.5	2.26
y2	2	12.5	2.26
y3	3	13.5	2.44
y4	4	22	3.98
y5	2,3,4	22	3.98
y6	1,3,4	22	3.98
y7	1,2,4	21	3.80
y8	1,2,3	14	2.53
y9	1,2,3,4	22	3.98

Table A1.2 Sensitivity analysis for the induced micromotion simulation. Outputs obtained for each simulation (y0-y9) are reported. Factors 1 to 4 are set at either their lower or higher value. Factor 1: Fibroting micromotion threshold; Factor 2: debonding tensile strength; Factor 3: debonding shear strength; Factor 4: friction coefficient.

Supplementary information 2

A2. Effect of the gap discretization step

In this analysis, the effect of the gap discretization step on the calibration results was evaluated. The calibration procedure was run two times using the same initial contact configuration, however the gap distribution obtained from experimental data was discretized and sampled with 10 µm and 5 µm binning, respectively. Similarly, the gap step was updated after each iteration using a discretization step of 10 µm and 5 µm, respectively. Gap threshold was fixed at 80 µm for this analysis.

The number of iterations needed for convergence was higher with a step of 5 µm compared to the 10 µm case (Figure A2), as well as the computational time. As reported in the previous analysis, final BIC percentage converged to the same value (Figure A2). Therefore, a gap step of 10 µm was considered sufficient and used in further analyses.

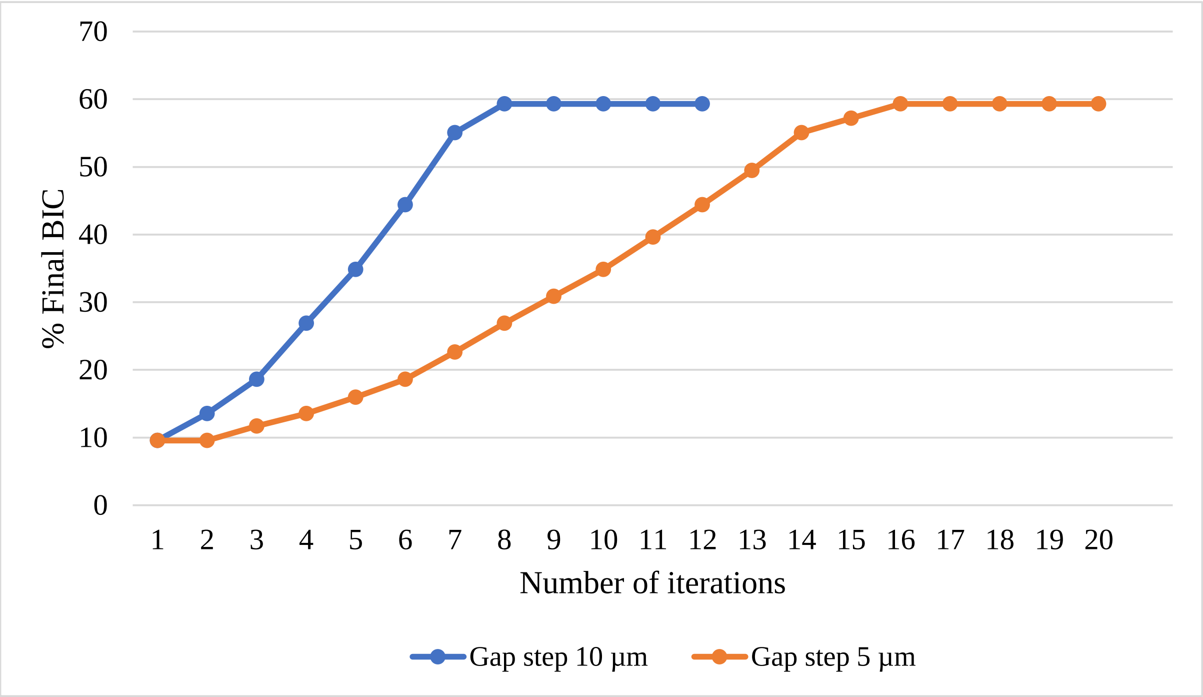

Fig. A2 Convergence plots of the Finite State Machine calibration. Final BIC percentage converged after 12 and 20 iterations, using a gap discretization step of 10 µm and 5 µm respectively.

Supplementary information 3

A3. Effect of the initial contact configuration

In this analysis, the effect of a variation in the input contact configuration on the predicted final BIC [%], and consequently on the gap threshold value, was evaluated. Ten different initial contact configuration distributions were randomly generated based on the distribution of bone-to-implant gaps measured experimentally, and used as input for the calibration procedure. Variations in the predicted final BIC were minimal (SD < 3%, Table A3).

	Final BIC [%]		
	**Gap threshold = 50 µm**	**Gap threshold = 70 µm**	**Gap threshold = 80 µm**
Configuration 1	34.84	48.14	55.59
Configuration 2	32.18	46.81	51.59
Configuration 3	34.84	55.05	59.31
Configuration 4	38.3	53.46	58.51
Configuration 5	32.71	49.47	56.65
Configuration 6	34.31	50.8	57.45
Configuration 7	38.03	51.86	57.18
Configuration 8	35.11	52.39	57.18
Configuration 9	38.56	52.39	57.98
Configuration 10	33.78	48.13	55.59
**Average [%]**	**35.27**	**50.85**	**56.7**
**Standard Deviation [%]**	**2.29**	**2.65**	**2.14**

Table A3 Final BIC [%] obtained with 10 different initial contact configurations (distributions of gap values).

References

[1] S. C. Cotter, “A screening design for factorial experiments with interactions,” *Biometrika*, vol. 66, no. 2, pp. 317–320, 1979, doi: 10.1093/biomet/66.2.317.
